# Young adult patients’ experience of living with mechanical circulatory support: A phenomenological hermeneutical study

**DOI:** 10.1002/nop2.247

**Published:** 2019-02-21

**Authors:** Maria Lachonius, Karl Hederstedt, Åsa B. Axelsson

**Affiliations:** ^1^ Institute of Health and Care Sciences Sahlgrenska Academy at University of Gothenburg Gothenburg Sweden; ^2^ Department of Cardiology Sahlgrenska University Hospital Gothenburg Sweden

**Keywords:** heart failure, mechanical circulatory support, patient’s experience, phenomenological hermeneutical, self‐efficacy, the lived body

## Abstract

**Aim:**

To describe young adult patients’ experiences of living with a mechanical circulatory support (MSC) as a bridge to heart transplantation and impact of self‐efficacy.

**Design:**

A qualitative and explorative interview study.

**Methods:**

Eight interviews with adult participants were conducted and analysed using the phenomenological hermeneutical method.

**Results:**

An overall theme, “Navigating from helplessness to feeling strong in the new reality,” and three themes were identified: “Feeling homeless in a changed reality” describes the experience of suddenly falling ill and the loneliness caused by the disease; “Finding my own inner resources” shows that the interviewees found the strength to fight for their lives and began to regain control of their situation; and “Adapting to my new reality” describes the importance of finding strength from others and being able to see MCS as a friend providing respite from the disease. Self‐efficacy beliefs play a significant role in the process that the participants went through.

## 1 INTRODUCTION

The incidence of heart failure is increasing, even among adult patients in the younger age group 18–44 years (Barasa et al., 2013). In some cases, the disease develops and becomes so serious that the patient becomes a candidate for heart transplantation. As the availability of organs is limited, the waiting period for a transplant can be long. Life‐threatening heart failure may develop, and the patient may need mechanical circulatory support (MCS) to survive until heart transplantation (Ozbaran et al., 2013). To be treated with MCS in the form of a heart pump system that supports both left and right ventricles (a biventricular assist device or BiVAD) means living with a conspicuous mechanical device attached to the body. The pump chambers are outside the body and cannot be concealed, and the device is thus an ever‐present attachment to the patient. Therefore, it is of interest to explore how relatively young patients treated with MCS experience their changed body image and the impact of self‐efficacy on their ability to manage the new situation.

## 2 BACKGROUND

A strong indication for MCS treatment is when a patient with congestive heart failure is judged to be in function class IV according to the New York Heart Association classification. In spite of optimal drug treatment, these patients are expected to have a poor prognosis for the progression of the disease, with a 1‐year mortality rate of approximately 50% (Lund, Matthews, & Aaronson, 2010). Patients treated with MCS as a bridge to transplant survive to a greater extent: 71% of those with MCS survived to transplantation compared with 33% with optimal drug treatment (Frazier et al., 2001).

Severe biventricular heart failure is a very serious condition, as illustrated by the survival rate in the American Interagency Registry for Mechanically Assisted Circulatory Support database (INTERMACS). Patients treated with BiVAD had a significantly worse 1‐year survival rate compared with those treated with a left ventricular assist device (LVAD) (55% vs. 80%) (Kirklin et al., 2015). Sahlgrenska University Hospital, Sweden, on the other hand, has shown a similar survival rate between BiVAD and LVAD groups (86% vs. 90%) (Bartfay et al., 2017).

Previous qualitative studies on MCS treatment describe how patients during MCS were struggling to adapt on various levels to their new life situation (Casida, Marcuccilli, Peters, & Wright, 2011; Chapman, Parameshwar, Jenkins, Large, & Tsui, 2007; Savage & Canody, 1999) and to their changed self‐image and body image (Chapman et al., 2007; Hallas, Banner, & Wray, 2009; Marcuccilli, Casida, & Peters, 2013). Managing daily activities was important, giving a sense of normality and control (Hallas et al., 2009; Marcuccilli et al., 2013). Further, Casida, Wu, Abshire, Ghosh, and Yang (2017) found that adherence to MCS care instructions correlated positively with self‐efficacy beliefs (*r* = 0.68) and general quality of life (*r* = 0.40). The above‐mentioned studies all had a mixed‐age patient population.

According to our experiences, the care of patients treated with MCS often has a focus on medical, technical or practical issues, but the healthcare professionals also need to focus on supporting and strengthen patients in an optimum way. It is important because a deep inability to cope with various life events, or to exercise control over stressful, persistent situations, is for a person the greatest obstacle to being able to adapt to an emerged situation (Benight & Bandura, 2004). Being treated with BiVAD could be considered as such a situation and self‐efficacy could be considered to play an important role in how patients treated with MCS endure their situation.

To our knowledge, there are no qualitative studies published describing younger adult patients’ experiences of living with MCS in the form of BiVAD. Further, there are few studies exploring how patients treated with MCS experience their changed body image and the impact of self‐efficacy to manage the new situation.

The aim of this study was therefore to describe these younger patients’ experience while they were living with MSC. Thus, the research question is as follows: What are the experiences from younger patients who have lived with MSC, while waiting for transplantation.

## 3 METHODS

### 3.1 Design

**Figure 1:**
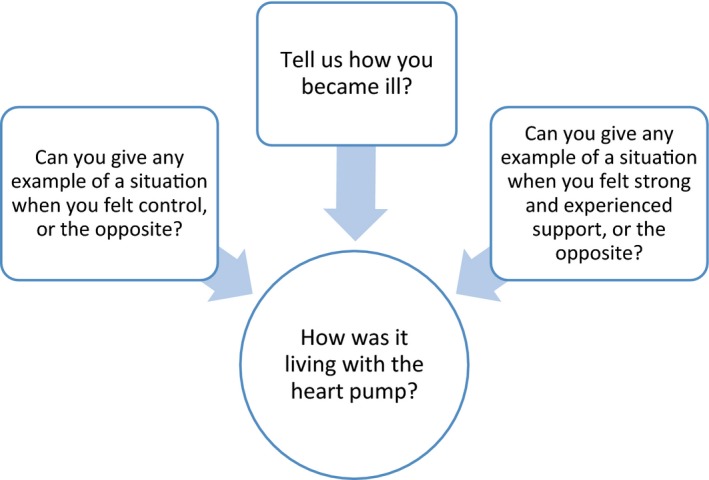
The relationship between the four questions in the question guide

A qualitative interview study with a consecutive sample was conducted to capture the patients’ experiences. An interview guide with four open questions covering the purpose of the study was designed. The four questions enabled the participants to narrate their experience of living with MCS (Figure 1). This manuscript was prepared according to consolidated reporting guidelines for qualitative research (Tong, Sainsbury, & Craig, 2007).

### 3.2 Settings and patient selection

All participants were recruited from a university hospital which is the only centre in Sweden to treat severe biventricular heart failure with BiVAD, in the form of a paracorporeal pump with blood chambers outside the body. A consecutive selection was made, allowing all patients between the ages of 18–44 years, between November 2013–February 2017, to be included. The age range 18–44 years was chosen according to the increasing incidence of heart failure in this age group (Barasa et al., 2013).

Eight participants were recruited consecutively (Table 1). Their biventricular heart failure had been very serious, with an ejection fraction between <10–20%. All participants were initially contacted and informed about the study when they had started their BiVAD treatment and while they were still in hospital care. BiVAD treatment had to be completed and the heart transplantation carried out before inclusion in the study was possible. At least 1 month after the transplantation, they were contacted again; all received written information about the study and all accepted participation. The interviews were conducted within 1 year of the heart transplant.

**Table 1:** Patient characteristics

Sex	Age	Days on MCS
Male	35	172
(*N* = 2)	18	81
Mean	26.5	126.5
Female	20	137
(*N* = 6)	22	101
	23	163
	42	168
	28	99
	34	139
Mean	28	134.5

Diagnosis	*N*	
(*N* = 8)		
DCMP	6	
DCMP, P P	1	
Myocarditis, P P	1	

Note

DCMP: dilated cardiomyopathy; MCS: mechanical circulatory support; P P: post‐partum.

### 3.3 Data collection

Eight face‐to‐face interviews were conducted. The participants had been offered the option of being interviewed at home, but all chose to be interviewed at the hospital. The interviews lasted between 30 min–2 hr. In one case, a parent was present without participating in the interview. Two of the authors (KH, ML) were present at all but one of the interviews. One of the authors had the main responsibility for the interview, and the other acted as assistant with the opportunity to ask supplementary questions at the end of the interview. The main responsibility varied. To ensure rigour, the participants were allowed to describe their experiences without interruptions or interpretations from the authors. The interviews were digitally recorded and notes were taken during the interviews.

### 3.4 Data analysis

The phenomenological‐hermeneutic method was chosen for the analysis (Lindseth & Norberg, 2004). The interviews were transcribed verbatim and to assure validity, the transcripts were read parallel with listening to the recordings. The transcriptions were then read several times to capture the whole meaning. This initial understanding of the text was formulated into a naïve reading. In the subsequent structured analysis, 396 meaningful units were identified in the text, which were reflected against the naïve understanding. These meaningful units were condensed by highlighting the essence of the statement. They were sorted into subthemes and then collected under themes. Then a main theme could be identified. The main theme and themes were compared with the naïve reading and validated it. Finally, the results were critically considered in relation to the literature (Lindseth & Norberg, 2004).

### 3.5 Ethical considerations

This study design fulfils the Declaration of Helsinki on ethical principles for medical research involving human subjects (World Medical Association, 2013). The research plan was approved by the Regional Ethical Review Board of Western Sweden (approval number DKr 458‐14). Written consent for participation was obtained from the interviewees after both oral and written information about the study was given, as well as guarantees of confidentiality when publishing the study results.

## 4 RESULTS

**Figure 2:**
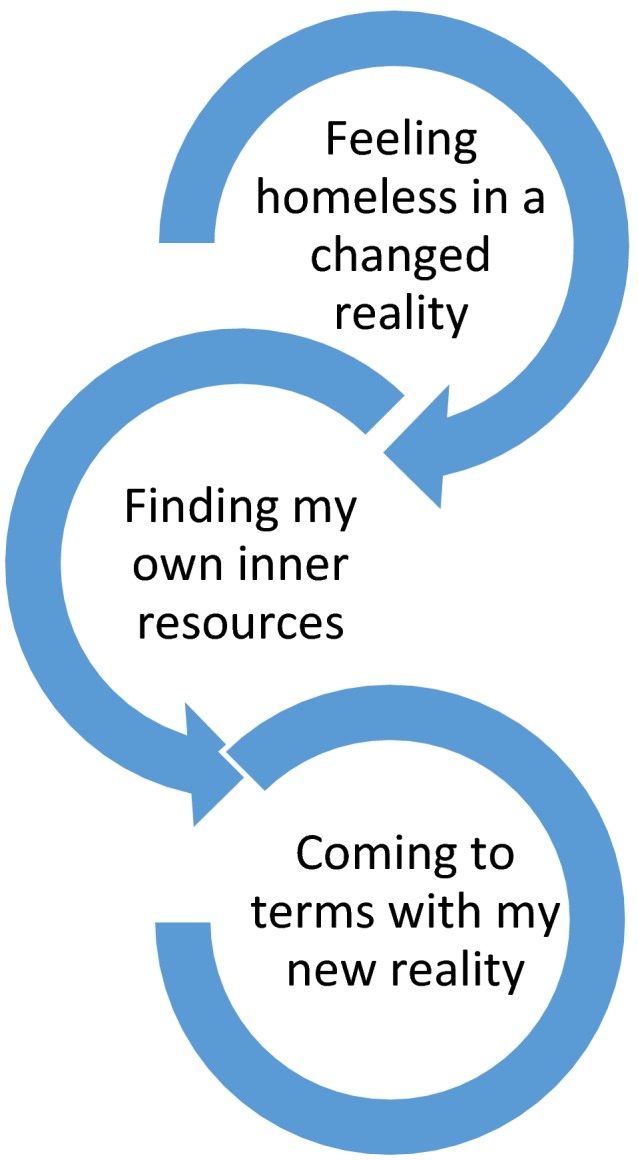
Main theme describing the process of navigating from helplessness to feeling strong in a new reality

The data analysis resulted in an overall theme, three themes and 16 subthemes (Table 2). The overall theme, *Navigating from helplessness to feeling strong in a new reality,* describes the process that the interviewees went through in their changed reality, while they were treated with MCS (Figure 2). The patients are referred to in the quotes below as P1–P8.

**Table 2:** Main theme, themes and subthemes

Main theme: Navigating from helplessness to feeling strong in a new reality		
Feeling homeless in a changed reality	Finding my own inner resources	Coming to terms with my new reality
A sudden change in life	Facing the disease	Adjusting to a changed life
Lost empowerment	Daring to surrender	Being strengthened by others
An invaded body	Managing to endure it	Seeing the pump as a friend
Being let down by my weak body	Finding my inner strength	Respite from the disease
The loneliness of the disease	Feeling confident	
Life in the slow lane	Regaining control of my own situation	

### 4.1 Theme 1: Feeling homeless in a changed reality

The first theme describes the interviewees’ experiences of sudden onset of the disease. They experienced a range of emotions that needed to be processed. They had become powerless because they were dependent on others. Most of the interviewees experienced their own body as strange and invaded. It had let them down, and they mourned their sick heart. The interviewees felt lonely and different. Life was going on around them, but their own life was running in the slow lane.

#### 4.1.1 A sudden change in life

The interviewees described the shock on waking up to discover that they had a life‐threatening illness. It was frightening, and seven out of eight described how they felt both anger and a sense of meaninglessness about their situation: “When I woke up, I didn't understand at first that it was true, because I thought I was perfectly well and my heart was great” (P3). All interviewees except one had started the MCS treatment with a BiVAD during the intensive care period without being able to participate in the decision to insert the heart pump. They felt a sense of unreality, and some interviewees felt like they were in a nightmare when they found the heart pump attached to their body: “I panicked of course, because I didn't know if it was for real, so I tried to pull everything off me” (P4).

#### 4.1.2 Lost empowerment

The interviewees were dependent on others and on MCS for their survival. They experienced a real threat of dying, and they were aware of the fragility of life. They were only alive thanks to the MCS treatment. The interviewees had to surrender to the healthcare professionals, with no room for their own choices or influence: “You felt very locked in... You were hooked up to a machine that kept you alive” (P4). Most of them felt powerless: “It also makes you panic when you don't have any control and you can't move, all power lies in everyone else's hands” (P3).

#### 4.1.3 An invaded body

Most interviewees experienced their own body as alarmingly different when they woke up after a long period of intensive care: “I was really upset every time I looked in the mirror so I put it off as much as I could” (P7). Some interviewees were disgusted by their body and were ashamed of their changed appearance: “I think it was quite disgusting to have such a thing. It's these tubes that make it look so brutal I think. It's just not me. I don't want to be associated with it” (P3). Some interviewees experienced panic attacks over the tubes that connected the pump to their body. They were constantly aware of the presence of the pump because it had invaded their body.

#### 4.1.4 Being let down by my weak body

The interviewees were aware that it was not their real heart keeping them alive; they were dependent on an artificial heart to survive. One interviewee expressed feelings of guilt for being alive, while another felt humiliated by her body's failures. They felt let down by their own weak body: “Why shouldn't I have the same right to live as everybody else... why am I less worthy than others because I got sick, because my heart wasn't strong enough to help me?” (P4). Most of the interviewees mourned their sick heart: “It still grieves me that my heart doesn't work” (P3). Some of them expressed ambivalence about needing a heart transplant: “I didn't want to have this, I just wanted it to be whole again but that's not the way it turned out” (P8).

#### 4.1.5 The loneliness of the disease

Living with MCS meant a feeling of being alone and looking different: “All the while I went around with the pump I felt, you know, this isn't me... I didn't want people to accept me like that because it wasn't me. That wasn't how I wanted to live” (P4). The pump could not be concealed and the constant noise it emitted also attracted unwanted attention: “You don't want to go out with it because everyone asks, what is that…? Then I think no, I'll just lock myself away” (P1). “You get very many reactions that you have to deal with because some people think it's disgusting” (P3). The loneliness of the illness became especially evident in the youngest interviewee, who expressed the difficulty of handling a crisis at such a young age.

#### 4.1.6 Life in the slow lane

Life during MCS treatment meant limitations in daily life. The interviewees could adapt to some situations, whereas others had to be completely excluded. For example, those who were parents could not take care of or be close to their children. They always lived life according to the limitations imposed by the pump treatment. The pump was a hindrance, and they were physically connected to it, shackled in their own body. They did not dare to make plans for the future: “You don't dare to plan, planning is hard” (P5). One interviewee expressed it as standing outside his own life: “You see and are there when everyone else is doing things, [you see] how the world continues to be, but you are not there taking part in it. You are sitting there like a spectator on the substitutes’ bench” (P4).

### 4.2 Theme 2: Finding my own inner resources

The second theme describes how the interviewees, after processing their feelings, began to accept their situation by facing the disease. They placed their trust in the healthcare professionals, and they focused on other things than the MCS treatment. The interviewees found their inner strength to endure their situation and fight to live. It was important to experience a sense of regaining control over the situation.

#### 4.2.1 Facing the disease

Knowing that the MCS treatment was vital meant that the interviewees could meet their illness and accept their changed reality: “It's better to have some limits than to be unable to do anything because you aren't alive” (P7). This also meant that most of the interviewees could see the MCS treatment in a more positive light: “I'd have been dead otherwise, that's a fact, I had a positive feeling about the pump” (P8). When the interviewees had begun to accept their changed reality, it also meant that they accepted the fact that they had become so seriously ill. Then, their grief over their own weak body could diminish: “I didn't feel my life was worth living but in time I understood that it definitely is” (P4).

#### 4.2.2 Daring to surrender

As time went by, most of the interviewees felt trusting and secure in the knowledge that the healthcare professionals had control over the situation: “You were confident that the hospital staff knew what they were doing, you know, that you could put your life in their hands” (P2). The interviewees developed a more pragmatic attitude to their life situation: “When I went for the heart transplant, it was like, yes but if I die here on the operating table, I can't think of any better way than that” (P5). They surrendered themselves and let go and realized that by daring to surrender, their well‐being could increase.

#### 4.2.3 Managing to endure it

The period of MCS treatment meant to be able to suffer through the situation. Knowing that the MCS treatment was temporary and that they would at some point get a heart transplant was important, as was living in the present, given their uncertain future: “I felt things were pretty fragile, so I tried to also live for the moment and try to live as well as possible with the pump” (P3). To endure this period, the interviewees tried to keep themselves occupied: “You kind of divide the day up, there's a new medicine... there's something good on TV... physiotherapy and visits” (P8). It was also important not to get bogged down but to focus on one thing at a time and think positively. In this way, most of them could bolster their own ability to handle their life situation.

#### 4.2.4 Finding my inner strength

The interviewees described how they eventually decided to fight to survive. They felt determined, not least those who were parents and must continue to exist for their children: “It took a while before I felt I really wanted to fight and get through this, you know, it took a few days for me to feel that way” (P2). Becoming as strong as possible before the heart transplantation was important. They found an inner strength, and they decided that they could survive: “The doctor comes in and says, your heart is fragile, you only have 10% capacity and we don't know how it will go. Then you feel like okay it's so... then I'll do the last thing I can” (P4).

#### 4.2.5 Feeling confident

The interviewees could gradually feel hope and confidence for the future. Some of them felt confident that a new heart would come, especially when they were registered as a candidate on the transplant waiting list: “This is not about the rest of my life... my heart will come soon” (P6). Most of the interviewees could see that a normal life was waiting for them after the heart transplant. However, one interviewee hoped that his own heart would recover: “I still hoped I wouldn't have to have a transplant, I hoped that for ages” (P8). They stressed the importance of seeing progress, as well as being able to trust in their own ability: “This is going to work out, it will be alright. And that was what made me strong” (P1).

#### 4.2.6 Regaining control over my own situation

Getting control over certain parts of everyday life was important to the interviewees. In this way, they could feel that they had influence over their daily lives, which strengthened their confidence in their own ability: “What I felt and what I could decide was how much exercise I had and how much I tried to get up and suchlike” (P2). It was significant when their physical strength improved. Setting realistic goals gave an important sense of regaining control of their situation: “You do a job, set a goal, I will get there because I want to, because, I knew it was good for me to think in that way” (P5).

### 4.3 Theme 3: Coming to terms with my new reality

The last theme describes the interviewees’ practical adaptation to their changed life situation. To reduce the loneliness of the disease and to come to terms with their new reality, they found it important to be strengthened and supported by those around them. It was also important to trust the pump and see it as a friend. Then, they could relax and get respite from the disease.

#### 4.3.1 Adjusting to a changed life

After facing the disease and beginning to accept their changed reality, the interviewees found it possible to adapt to a changed life. They got used to the pump and learned to manage it in their everyday lives: “Then after a while, when you got into it, there was no problem then, though it was still hard, but you could manage it” (P1). They adapted to the limitations that MCS imposed on their everyday life: “...I didn't dare live that way and go out and meet people, I'd rather hide myself away and wait. But that didn't work either, so I had to live in the there and now” (P5).

#### 4.3.2 Being strengthened by others

To be strengthened by their close friends and relatives, as well as by their fellow patients and the healthcare professionals, was very important to the interviewees in to feel at home in their new reality: “Then I met some others who were transplanted and then I felt, okay there is something better to come” (P3). Family support was invaluable. Being understood as a unique individual also meant that the interviewees felt stronger. They felt affirmed when the healthcare professionals genuinely listened to them. To feel this support, it was very important that they were truly present and empathic: “To listen, like, when you, if you have something to say, that they really take the time to listen” (P2).

#### 4.3.3 Seeing the pump as a friend

The interviewees reported that feeling that they trusted the pump and seeing it as a friend helped them to relax: “But then I tried to feel safe, that I'm safer now than before the cardiac arrest, because then I was like a ticking bomb. It works, it sounds good and it will make an alarm sound if it isn't working...” (P3). Seven out of eight interviewees described that they had come to terms with the pump. Some interviewees experienced some sort of normalization of their life situation: “I forgot it sometimes. There were times when I like, oh yes, I'll have the pump with me as well, so it became like a part of me, that you even forgot all about it” (P5). By feeling trust and confidence in the pump, they felt more at home in their new reality.

#### 4.3.4 Respite from the disease

Humour made it possible to find respite from the disease by joking and chatting with the family or the nurses, as well as being able to perform and enjoy everyday activities: “I was in the computer, in my own world” (P7). The interviewees were anxious to know that there was a regular life, which could mean friends coming to dinner. By forgetting the MCS treatment for a while, they felt a sense of normality and freedom: “I came up with a trick. It was to wear earphones and headphones, then I could disappear for a few minutes” (P4). “When you sleep, you're free... but as soon as you wake up... then I heard it, of course” (P8).

### 4.4 Comprehensive understanding and discussion

The overall theme of this study: 'Navigating from helplessness to feeling strong in a new reality', demonstrates the importance of self‐efficacy in the process that patients undergo to be able to accept MCS treatment in their changed reality. The fact that the interviewees felt that they could independently manage and have control over certain aspects of their daily lives was necessary for their self‐efficacy to increase. Previous research has shown a negative correlation between overprotection from significant others and self‐efficacy and well‐being (Joekes, Van Elderen, & Schreurs, 2007). Support given to patients with MCS should enable them to feel a growing self‐efficacy through an increased degree of independence. Further, patients on MCS have been found to be prepared to be discharged and continue their care in an outpatient clinic once they had learned the self‐management regime during their hospitalization (Casida et al., 2017). Marcuccilli, Casida, Bakas, and Pagani (2014) found that patients and caregivers can be strengthened in their confidence in themselves by the professional healthcare team before discharge from hospital, to enable them to make the necessary adjustments to their lifestyle. This may facilitate the transition to continued care in a cardiology outpatient clinic, which implies for the patient a step towards a sense of normality. Further, from a socioeconomic perspective, a hospital bed is released for other patients in need of cardiologic specialist care, which is important when care facilities are limited.

The first theme, “Feeling homeless in a changed reality,” describes how the interviewees suddenly found themselves in a changed reality when they had abruptly developed a life‐threatening condition. Their own body, which had let them down, was frighteningly unfamiliar. This corresponds well with Merleau‐Ponty's philosophical description of the body as our access to the world, which means that, if illness strikes, the world changes (Merleau‐Ponty, 2012). Merleau‐Ponty (2012) postulated that a changed body image is a traumatic experience that can trigger a suppression mechanism, where the situation that has arisen is not accepted. This reasoning is confirmed by the fact that the interviewees experienced their own body as disgusting and invaded and felt that they did not want to be associated with the heart pump. As Merleau‐Ponty (2012) describes, their being in the world was changed such that the fusion of body and soul was altered. Their unique personal life‐world had ceased to exist. That the interviewees’ unique personal life‐world ceased to exist is consistent with losing empowerment and experiencing the loneliness of the disease. The disease had interrupted their everyday life and they experienced homelessness, in that their belonging in the world ceased. This is confirmed by Svenaeus (2011), who described how being seriously ill causes an “unhomelike” and uncanny experience of being in the world.

The second theme, “Finding my own inner resources,” describes how the interviewees, by processing what had happened, could gradually begin to accept their changed reality. They faced the disease and dared to surrender themselves to their treatment. This finding is supported by Merleau‐Ponty's (2012) reasoning that the person's own lived body must connect to and accept the actions that are taking place around it. Then, the person can perceive the emerging situation as manageable.

By finding their inner strength and feeling resolute in this difficult situation, the interviewees could regain a sense of control. Their self‐efficacy increased when they could see their progress and they managed to get through the time on MCS. Benight and Bandura (2004) describe the importance of the protective effect of self‐efficacy in response to threats and traumatic events. When the feeling of being able to control their changed life situation increased, the interviewees experienced the situation as less threatening. They could rely on their own ability to handle the situation, demonstrating the importance of high self‐efficacy in managing life with MCS.

Finally, the third theme, “Coming to terms with my new reality,” indicates that the loneliness of the disease could be alleviated when the interviewees were confirmed by others. Living a normal life meant being able to rest from the disease. The interviewees had adapted to their new reality, which was facilitated by their increased self‐efficacy. The interviewees could see the heart pump as a friend, as they accepted the pump as lifesaving. They re‐evaluated and expanded their own world to include the pump. The pump was not alien but rather to a certain extent a part of the lived body. Merleau‐Ponty (2012) has described this process as an extension of the body and thus the lived body.

The interviewees in our study underwent a profound process to manage their changed reality, which has also been explored in a LVAD population by Standing, Rapley, MacGowan, and Exley (2017). Previous research has illustrated that parts of the pump can become an extension of the patient's body and that the interface between the body and the MCS changes when the body adapts to the MCS (Sandau, Hoglund, Weaver, Boisjolie, & Feldman, 2014). This reflects the proposition that the pump became part of the lived body. However, our interviewees did not fully see their MCS as part of the body, which may be due to the fact that their BiVAD was a larger pump than other heart pump designs, which gave a significantly larger bodily change than with other MCS equipment. The reaction to the bodily change could also reflect the fact that our interviewees were relatively young. Our study therefore suggests that a BiVAD could never be fully accepted as a part of the lived body.

Our results highlight the importance of patients being able to accept the pump treatment to become accustomed to their new existence. To be able to endure in their new reality is also an important finding, which has not been described previously in younger adult patients with MCS and with BiVAD in particular. All our findings reinforce the importance of supporting this patient group in an optimum way.

### 4.5 Limitations and strengths

One limitation of the study is that few participants were recruited and during a period of 39 months. It can be argued that it is a weakness of present study that it has elapsed a long time between the inclusion of the first and last patient. However, no structural changes about the care of this group of patients were done during this time. Since all patients who met the inclusion criteria for the study were included in the current data collection period, this illustrates that younger patients as in this study's age range and in need of advanced therapy for severe biventricular heart failure constitute, is a rare patient group. It also reflects that BiVAD treatment is only given when absolutely no other options are eligible. The eight completed interviews gave a rich source of data and the interviewees had often similar experiences and insights, which indicates that the data collection was sufficiently extensive for a narrative analysis, searching for phenomenal variations (Sandelowski, 1995).

Trustworthiness and authenticity were strengthened by the choice of method, as the phenomenological hermeneutical methodology allows the essence of the interviewees’ experiences to come to light. The accuracy required in the analysis to both understand and interpret the lived experience in each interviewee's story strengthens the study's credibility (Lindseth & Norberg, 2004), despite the limited selection of patients.

The findings show a consistency that confirms the naïve reading formulated in the initial analysis, which strengthens the authenticity of the study. The result was verified by a comprehensive understanding of the literature, which demonstrated coherence that strengthens our findings (Lindseth & Norberg, 2004).

## 5 CONCLUSIONS

Patients’ living with MCS needs to gain an increased belief in their ability to take control over their changed reality. This study shows the importance of self‐efficacy belief in the process that younger adult patients undergo to be able to accept the treatment and their changed reality, while they are living with MCS. Self‐efficacy can increase if patients independently manage and have control over certain aspects of their activities of daily living. The healthcare team needs to be aware of the importance of strengthening patients’ self‐efficacy belief, the significance of the lived body and how this abruptly changed life situation affects the patient's entire reality. Through in‐depth knowledge, the healthcare team can meet and support patients based on their individual circumstances and life‐world. A structured person‐centred approach in the care of these patients may facilitate the strengthening meeting between the patient and the healthcare team and therefore enhance the patients’ confidence in their own ability to grow. These issues need to be given both time and space in the daily care schedules. Further research is needed to evaluate which kind of support is experienced to have the best effect.

## CONFLICT OF INTEREST

None to declare.
